# Dysregulated PJA1-TGF-β signaling in cancer stem cell–associated liver cancers

**DOI:** 10.18632/oncoscience.522

**Published:** 2020-11-30

**Authors:** Jian Chen, Julian A. Gingold

**Affiliations:** ^1^Department of Gastroenterology, Hepatology, & Nutrition, The University of Texas MD Anderson Cancer Center, Houston, TX, USA; ^2^Sandhill Therapeutics, Inc., Dallas, TX, USA; ^3^Montefiore Medical Center, OB/GYN and Women’s Health, Bronx, NY, USA

**Keywords:** TGF-β signaling, PJA1 E3 ligase, liver cancer stem cells, hepatocellular carcinoma

## Abstract

The transforming growth factor beta (TGF-β) signaling pathway plays important roles in cell differentiation, stem cell modulation, organ lineage, and immune suppression. TGF-β signaling is negatively regulated by the ubiquitin–proteasome pathway. Although mouse models of cancer arising from a defective TGF-β pathway clearly demonstrate the tumor-suppressive role of TGF-β, the underlying mechanism by which a defective TGF-β pathway triggers liver cancer development is poorly understood. This review summarizes key findings from our recent studies connecting TGF-β to hepatic oncogenesis and highlights the vulnerability of TGF-β signaling to PJA1-mediated ubiquitination. TGF-β, together with the chromatin insulator CCCTC-binding factor (CTCF), epigenetically and transcriptionally regulate tumor promoter genes, including IGF2 and TERT, in TGF-β–defective mice and in human liver cancers. Dysfunction of the TGF-β–regulated SPTBN1/SMAD3/CTCF complex increases stem cell–like properties in hepatocellular carcinoma (HCC) cells and enhances tumorigenesis in tumor-initiating cells in a mouse model. PJA1, a novel E3 ubiquitin ligase, is a key negative regulator of TGF-β signaling. PJA1 overexpression is detected in HCCs and is sufficient to suppress SMAD3- and SPTBN1-mediated TGF-β tumor suppressor signaling, promoting HCC proliferation. Dysregulated PJA1-TGF-β signaling activates oncogenic genes and promotes tumorigenesis in human liver cancers. In addition, inhibition of PJA1 by treatment with E3 ligase inhibitors restores TGF-β tumor-suppressor function and suppresses liver cancer progression. These new findings suggest potential therapeutic avenues for targeting dysregulated PJA1-TGF-β signaling via cancer stem cells in liver cancers.

## INTRODUCTION

Hepatocellular carcinoma (HCC) is the solid tumor type with the fastest-rising incidence in the United States as well as the second-leading cause of cancer-related death worldwide [1, 3]. Survival rates for patients affected by HCC remain dismal, despite the development and approval of several targeted chemo- and immunotherapies. Sorafenib, a multi-target tyrosine kinase inhibitor, was the only systemic therapy for advanced HCC with FDA approval prior to 2016 [3]. Recently, three new multi-kinase inhibitors, lenvatinib [4], regorafenib [5], and cabozantinib [6], and an antibody-based VEFGR2 antagonist, ramucirumab, received FDA approval for advanced HCC [7]. Unfortunately, the median overall survival for patients treated with any of the above agents remains less than 15 months [8]. Thus, there is an urgent need for more effective and targeted therapies in this large HCC patient population.

The complexity of tumor initiation, progression, metastasis, and therapeutic resistance in liver cancer is deeply associated with tumor stem cell development, the tumor microenvironment, tumor immunity, and genomic alterations [9]. The two major causes for failure of liver cancer therapy are cirrhosis and drug resistance, both of which are potentially mediated through cancer stem cells [10]. The TGF-β pathway also plays a complex role in liver diseases including cirrhosis and liver cancer, where it variously exerts fibrogenic/proinflammatory, tumor suppressive, or pro-metastatic effects [11]. Mouse models with a defective TGF-β pathway clearly demonstrate the tumor suppressor role of TGF-β [12-15], although an underlying mechanism by which a defective TGF-β pathway supports liver cancer development has not been established. Clinically, high levels of TGF-β are associated with a favorable prognosis in early-stage cancers, supporting a primarily tumor-suppressive role for this signaling pathway; however, in advanced-stage or metastatic tumors, high levels of TGF-β are associated with tumor invasiveness and dedifferentiation, highlighting the context-dependence of TGF-β signaling effects even in liver cancers [16-19]. How TGF-β alterations initially inhibit liver cancer development but later exacerbate the malignancy’s aggressiveness through pro-oncogenic activities remains poorly understood. This review focuses on emerging mechanistic insights into the tumor suppressor role of TGF-β and its primary regulators in liver cancer development, particularly at its early stages.

## HCC stem cell signature is characterized by defective TGF-β signaling

**Figure 1:**
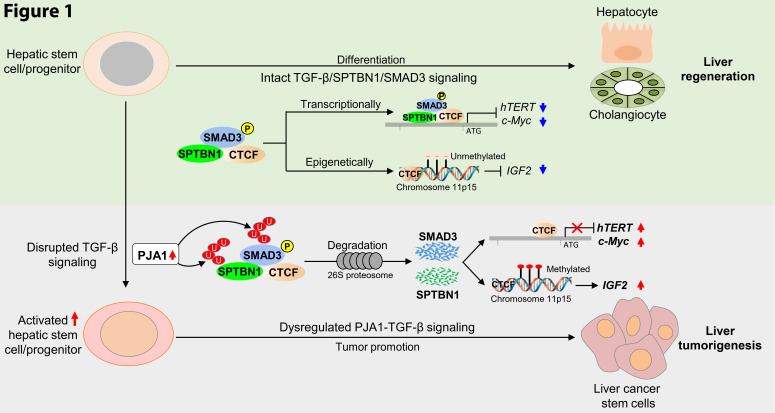
PJA1 suppresses SPTBN1/SMAD3-mediated TGF-β signaling in hepatic stem cells. PJA1 activity disrupts TGF-β signaling at multiple levels, including ubiquitination/degradation of SPTBN1 and p-SMAD3, down-regulation of the SPTBN1/SMAD3/CTCF complex, and promotion of tumor promoter genes IGF2, TERT, and Myc. Collectively, disrupted TGF-β signaling results in development of cancer stem cell-associated tumorigenesis. P, phosphorylated; U, ubiquitinated.

We previously showed that disruptions in TGF-β signaling, coupled with loss of the SMAD adaptor SPTBN1, resulted in the spontaneous development of multiple tumors and a high incidence of liver cancer in human fibroblast and mouse models [20]. TGF-β, together with the tumor suppressor CTCF, epigenetically and/or transcriptionally regulate tumor promoter genes, including IGF2, TERT, and Myc in TGF-β–defective mice and in patients with Beckwith-Wiedemann syndrome (BWS), a human stem cell disorder [20]. Dysfunction of the TGF-β–regulated SPTBN1/SMAD3/CTCF complex increases stem cell–like properties in HCC cells and enhances tumorigenesis in tumor-initiating cells in a mouse model (Figure 1) [20].

We analyzed the transcriptome sequencing data from 147 HCCs in The Cancer Genome Atlas (TCGA) for TGF-β pathway genes [21]. TGCA data indicated that genes directly associated with the TGF-β superfamily were consistently dysregulated (i.e., either elevated or suppressed) [21]. Patients demonstrating a defective TGF-β signature (low levels of TGF-β) experienced significantly poorer outcomes than those with an intact TGF-β signature (high and normal levels of TGF-β) (hazard ratio = 3.15, log-rank test p-value = 0.0027) [21].

There was variable expression of stem cell signatures across 9,125 samples from 33 tumor types, including 368 HCCs, in the TCGA database [22, 23]. Overall, low TGF-β pathway activity across the 33 tumor types was associated with a higher “stem cell index,” defined by expression of stem cell genes. The HCC samples in this data set were classified into high, intermediate, or low stem cell index tumors. There was a negative correlation between TGF-β pathway activity and stem cell–like character. These results indicate that impairment of TGF-β signaling may contribute to cancer stem cell–associated HCC development [22, 23].

In advanced HCC, high levels of TGF-β are associated with increased inflammation and tumor invasiveness. The oncogenicity of TGF-β signaling is bolstered by results from a recent phase 2 clinical trial, in which inhibition of TGF-β signaling using a TGF-β receptor 1 inhibitor increased survival of patients with advanced HCC whose tumors expressed high levels of TGF-β and alpha-fetoprotein from 9 months to 21 months [24]. The clinical efficacy of TGF-β pathway inhibition and relevance of TGF-β signaling to tumor progression demonstrates that the pathway carries the potential to mediate oncogenic as well as tumor suppressive effects.

Although this treatment modality demonstrated efficacy in patient tumors expressing high TGF-β, there are currently no therapeutic strategies for HCC patients with a defective TGF-β pathway (low levels of TGF-β). Therefore, our recent attention has been turned to identifying oncogenic mechanisms underlying a defective TGF-β pathway, in particular the potential for defective TGF-β signaling to promote liver cancer stem cell growth and survival.

## Increased expression of E3 ubiquitin ligase PJA1 in HCC promotes liver tumorigenesis by reducing TGF-β/SMAD3/SPTBN1 activity

We analyzed transcriptome sequencing data of 374 HCC patient samples in TCGA [22]. The amount of PJA1 mRNA was significantly increased in HCC compared with normal liver. An increase in PJA1 transcripts in HCC patients relative to levels in normal liver was also detected in the Roessler liver 2 data from Oncomine and the Wurmbach liver data from the Gene Expression Omnibus [25]. Analysis of PJA1 protein expression revealed increased levels in HCCs compared with normal livers [22]. Furthermore, TCGA data indicated that increased mRNA expression of PJA1 was associated with markedly reduced overall survival of patients with HCC, suggesting that high activity of PJA1 may confer a poor HCC prognosis.

Numerous E3 ubiquitin ligases have been identified to negatively regulate various components of the TGF-β pathway, and alterations of ubiquitin ligases been recognized in several cancer types [26-34]. TGF-β family members transduce signals through membrane serine/threonine kinase receptors and intracellular effectors from the SMAD family of proteins [35, 36]. Assembly of components for adequate SMAD signal transduction requires adaptor proteins, which tightly regulate SMAD nuclear translocation, receptor phosphorylation and degradation [35].

PJA1, a RING finger E3 ubiquitin ligase of the Praja family, promotes ubiquitination and degradation of multiple targets, including SMAD3, homeodomain protein Dlx5, polycomb repressive complex 2 proteins (PRC2), and Enhancer of zeste homologue 2 (EZH2) [37-39]. Although the expression of PJA1 is increased in some cancers, including glioblastoma and some gastrointestinal tract cancers [40, 41], detailed studies of PJA1 in HCC development and progression have not been conducted.

Recently, we identified the PJA1 as a protein that ubiquitinates SPTBN1 in a TGF-β–dependent manner [22, 40]. Accordingly, PJA1 E3 ligase activity regulates TGF-β signaling by controlling SPTBN1 abundance through ubiquitin-mediated degradation. We also found that PJA1-mediated ubiquitination of phosphorylated (p)-SMAD3 in HCC cells only occurred in cells exposed to TGF-β. These data indicate that PJA1 promotes the ubiquitination and proteasomal degradation of p-SMAD3, resulting in reduced activity of the TGF-β/SMAD3/SPTBN1 tumor-suppressing pathway in HCC cells (Figure 1). Thus, the PJA1 interaction with SMAD3 in HCC is TGF-β–dependent.

Knockdown of PJA1 by short-hairpin RNA (shRNA) significantly reduced colony formation in HCC cells and anchorage-independent growth of SNU475 and HepG2 cells [22]. Moreover, knockdown of PJA1 impaired tumor growth in a xenograft model of subcutaneously injected HepG2 cells in nude mice. Knockdown of PJA1 resulted in reduced numbers of Ki67-positive cells and increased numbers of cells positive for the apoptosis effector Caspase3, suggesting that PJA1 promoted HCC cell proliferation and protected against apoptosis. These data indicate that PJA1 functions as a tumor promoter and that reducing its activity in liver cancer cells impairs malignant phenotypes.

## PJA1 may promote liver cancer stem cell proliferation and liver metastasis in the context of defective TGF-β

To elucidate the mechanisms by which PJA1 regulates TGF-β signaling in cancer stem cell–associated liver tumorigenesis, we developed a novel mouse model using a hydrodynamic (rapid injection) delivery approach (PiggyBac Transposon System, System Biosciences, Inc.). By injecting a PJA1-encoding Sleeping Beauty (SB) transposon through the mouse tail vein, the plasmid primarily accumulates in the liver, allowing for selective overexpression of mouse PJA1 in hepatocytes [22]. This approach built upon our previously established models of IL6-mediated chronic inflammation in both wild-type and TGF-β defective (*Sptbn1*^+/-^) mice utilizing hydrodynamic injection of the IL6 gene every 2 weeks for 3 months [42].

A mouse PJA1-encoding SB transposon and control plasmid DNA SB transposase were injected hydrodynamically through the mouse tail vein in wild-type Black6 background mice twice per month for 3 months. Stable expression of PJA1 in the liver of wild-type Black6 mice after 3 months of tail vein injection was confirmed by immunohistochemical analysis, while PJA1 expression remained very low in mouse liver infected by control plasmid DNA.

We explored the effects of PJA1 overexpression on stem cell properties in wild-type Black6 and TGF-β defective (*Smad3*^+/-^) mice. At day 90, single-cell suspensions from the mouse livers were prepared for liver stem cell (LSC) isolation [42]. LSCs (CD133 positive cells) were isolated from each liver preparation by using a magnet-activated cell-sorting column with an antibody recognizing CD133. The cells were assessed for cell proliferation, anchorage-independent growth, and colony formation, and were injected into immune-compromised mice to evaluate for serial tumor formation and liver metastasis [22]. Our data demonstrated that LSCs from the PJA1-injected TGF-β-defective (*Smad3*^+/-^) mice showed a higher proliferation rate, increased Ki67 staining in cell culture, and exhibited increased cell transformation in soft agar compared with either PJA1-injected wild-type Black6 or the plasmid control-injected TGF-β-defective (*Smad3*^+/-^) mice. Interestingly, neither the LSCs from the control-injected TGF-β-defective (*Smad3*^+/-^) (n=6) nor the PJA1-injected wild-type Black6 mice (n=6) formed tumors or liver metastases when LSCs were injected subcutaneously into immune-compromised mice. However, 2 mice from total 6 mice injected with LSCs from the PJA1-injected TGF-β-defective (*Smad3*^+/-^) mice formed tumors and liver metastases, suggesting that increased PJA1 in the context of defective TGF-β signaling promotes liver stem cell properties and their transformation into cancer stem cells (Figure 1) [22].

## Targeting dysregulated PJA1-TGF-β signaling in liver cancers

We recently reported that TGF-β–deficient mice of various genetic backgrounds, including TGF-β signaling adaptor *Sptbn1*^+/-^ mice and double-knockout *Sptbn1*^+/-^/*Smad3*^+/-^ mice, develop multiple tumors, including HCC [20, 43]. These mice are characterized by macroglossia, cardiomegaly, renal hypertrophy, and conspicuous cytomegaly of the adrenal cortex [20, 43] and generally resemble the human BWS phenotype. Thus, genetic studies demonstrate that defective TGF-β signaling can phenocopy a human stem cell disorder.

SMAD3 and its adaptor protein SPTBN1 are increasingly recognized as potent regulators of liver cancer stem cell development. Our recent work indicated that PJA1 promotes the ubiquitination of SPTBN1 and p-SMAD3, resulting in reduced activity of the tumor-suppressing TGF-β/SMAD3/SPTBN1-dependent pathway in HCC cells [20, 40]. Although our data strongly support a key role for TGF-β signaling in suppressing liver cancer and highlight how PJA1 E3 ligase inhibits TGF-β signaling, the precise mechanism of dysregulated PJA1-TGF-β signaling and its role in the stages of HCC development remain unclear.

Cirrhotic livers precede HCC in 80%–90% of patients and are considered precancerous lesions [44, 45]. TERT promoter mutations have been reproducibly associated with cirrhosis and cirrhosis-associated HCC [45]. TERT promoter mutations were found in 6% of low grade dysplastic nodules and 19% of high-grade dysplastic nodules in cirrhosis, and were dramatically increased in early HCCs (61%) without additional recurrent HCC driver gene mutations. These tumor genetic studies demonstrate that TERT promoter mutation is one of the earliest recurrent somatic genetic alterations during the transformation sequence from liver cirrhosis to HCC [46-48]. TERT promoter mutations are associated with oncogenic CTNNB1 mutations [46, 47, 49] and epigenetic silencing of the tumor suppressor CDKN2A (p16INK4A) [9], suggesting that activated telomerase may cooperate with TGF-β signaling to initiate liver tumor formation.

Interestingly, our findings revealed that TGF-β, together with CTCF, epigenetically/transcriptionally regulate TERT [20]. Dysfunction of TGF-β–regulated CTCF increases stem cell–like properties in HCC cells and enhances tumorigenesis in tumor-initiating cells in a mouse model, suggesting that defective TGF-β/CTCF might cooperate in liver tumor initiation [14]. Importantly, PJA1 knockdown significantly increases binding of SMAD3 and SPTBN1 to the TERT promoter, thereby transcriptionally increasing TERT gene expression in HCC cells, suggesting that PJA1 may also regulate TERT activity through TGF-β and/or CTCF-mediated epigenetic regulation (Figure 1) [22].

In our ongoing studies, increased levels of PJA1 protein expression were detected in 100% of human liver cirrhosis and cirrhotic HCC patient samples. The strong expression of PJA1 even at the premalignant cirrhosis stage suggests that PJA1 might drive survival and proliferation of cancer progenitor cells with the potential to develop into HCC. Therefore, we hypothesize that PJA1 plays a central role in chronic fibrosis/inflammation in the liver and cancer stem cell–associated HCC development. These findings also highlight the clinical potential of using PJA1 inhibition to block liver fibrogenesis and possibly oncogenesis (Figure 1).

## CONCLUSION

Effective therapeutic strategies targeting HCC are urgently needed and may be achieved by identifying critical signaling pathways relevant in liver cirrhosis and the early stages of liver cancer.

Our genomic analyses of TGF-β in liver and other cancers indicate that genes directly associated with the TGF-β superfamily are recurrently dysregulated (either elevated or suppressed) [21, 50]. However, no therapeutic strategies for HCC patients with a defective TGF-β pathway (low levels of TGF-β) exist. Moreover, patients with tumors harboring a defective TGF-β signature have significantly worse outcomes than those with tumors with an intact TGF-β signature (high and normal levels of TGF-β) [21].

Since TGF-β signaling is recognized to drive the epithelial-mesenchymal transition and liver fibrosis progression toward advanced liver cancer, many drugs targeting TGF-β signaling in liver cancers are currently under investigation in clinical trials [51]. Despite successful outcomes of different anti-TGF-β approaches in cell culture and animal models, cancer clinical trials utilizing strategies to block TGF-β signaling have demonstrated poor or inconsistent results so far [51], suggesting a crucial yet underexplored context-dependent role of TGF-β signaling in human cancers. It is possible that the failure of many of these agents is a consequence of the underappreciated tumor suppressor functions of TGF-β signaling.

Recent mouse model studies have clearly demonstrated the oncogenic potential of disrupting both PJA1 and TGF-β signaling. The consequences of disruption of these genes are likely mediated through liver cancer-initiating cells and/or liver cancer stem cells. Identifying mechanisms to activate TGF-β expression by inhibition of its negative regulators, such as PJA1 E3 ligase, may broaden the usefulness of pathway–based therapies targeting TGF-β. In addition, future therapies may combine E3 ligase inhibition with TGF-β pathway activation to help overcome signaling roadblocks that evolve within tumors. We anticipate significant therapeutic advances to arise from future basic and clinical research exploring the dysregulated PJA1-TGF-β pathway in HCC.

## References

[1] Kulik L, El-Serag HB (2019). Epidemiology and Management of Hepatocellular Carcinoma.. Gastroenterology.

[2] Llovet JM, Montal R, Sia D, Finn RS (2018). Molecular therapies and precision medicine for hepatocellular carcinoma.. Nat Rev Clin Oncol.

[3] Llovet JM, Ricci S, Mazzaferro V, Hilgard P, Gane E, Blanc JF, de Oliveira AC, Santoro A, Raoul JL, Forner A, Schwartz M, Porta C, Zeuzem S, SHARP Investigators Study Group (2008). Sorafenib in advanced hepatocellular carcinoma.. N Engl J Med.

[4] Kudo M, Finn RS, Qin S, Han KH, Ikeda K, Piscaglia F, Baron A, Park JW, Han G, Jassem J, Blanc JF, Vogel A, Komov D (2018). Lenvatinib versus sorafenib in first-line treatment of patients with unresectable hepatocellular carcinoma: a randomised phase 3 non-inferiority trial.. Lancet.

[5] Bruix J, Qin S, Merle P, Granito A, Huang YH, Bodoky G, Pracht M, Yokosuka O, Rosmorduc O, Breder V, Gerolami R, Masi G, Ross PJ, RESORCE Investigators (2017). Regorafenib for patients with hepatocellular carcinoma who progressed on sorafenib treatment (RESORCE): a randomised, double-blind, placebo-controlled, phase 3 trial.. Lancet.

[6] Abou-Alfa GK, Meyer T, Cheng AL, El-Khoueiry AB, Rimassa L, Ryoo BY, Cicin I, Merle P, Chen Y, Park JW, Blanc JF, Bolondi L, Klümpen HJ (2018). Cabozantinib in Patients with Advanced and Progressing Hepatocellular Carcinoma.. N Engl J Med.

[7] Zhu AX, Kang YK, Yen CJ, Finn RS, Galle PR, Llovet JM, Assenat E, Brandi G, Pracht M, Lim HY, Rau KM, Motomura K, Ohno I, REACH-2 study investigators (2019). Ramucirumab after sorafenib in patients with advanced hepatocellular carcinoma and increased α-fetoprotein concentrations (REACH-2): a randomised, double-blind, placebo-controlled, phase 3 trial.. Lancet Oncol.

[8] Yarchoan M, Agarwal P, Villanueva A, Rao S, Dawson LA, Llovet JM, Finn RS, Groopman JD, El-Serag HB, Monga SP, Wang XW, Karin M, Schwartz RE (2019). Recent Developments and Therapeutic Strategies against Hepatocellular Carcinoma.. Cancer Res.

[9] Ally A, Balasundaram M, Carlsen R, Chuah E, Clarke A, Dhalla N, Holt RA, Jones SJ, Lee D, Ma Y, Marra MA, Mayo M, Moore RA (2017). Comprehensive and Integrative Genomic Characterization of Hepatocellular Carcinoma.. Cell.

[10] Nault JC, Ningarhari M, Rebouissou S, Zucman-Rossi J (2019). The role of telomeres and telomerase in cirrhosis and liver cancer.. Nat Rev Gastroenterol Hepatol.

[11] Principe DR, Doll JA, Bauer J, Jung B, Munshi HG, Bartholin L, Pasche B, Lee C, Grippo PJ (2014). TGF-β: duality of function between tumor prevention and carcinogenesis.. J Natl Cancer Inst.

[12] Muñoz NM, Upton M, Rojas A, Washington MK, Lin L, Chytil A, Sozmen EG, Madison BB, Pozzi A, Moon RT, Moses HL, Grady WM (2006). Transforming growth factor beta receptor type II inactivation induces the malignant transformation of intestinal neoplasms initiated by Apc mutation.. Cancer Res.

[13] Romero-Gallo J, Sozmen EG, Chytil A, Russell WE, Whitehead R, Parks WT, Holdren MS, Her MF, Gautam S, Magnuson M, Moses HL, Grady WM (2005). Inactivation of TGF-beta signaling in hepatocytes results in an increased proliferative response after partial hepatectomy.. Oncogene.

[14] Bardeesy N, Cheng KH, Berger JH, Chu GC, Pahler J, Olson P, Hezel AF, Horner J, Lauwers GY, Hanahan D, DePinho RA (2006). Smad4 is dispensable for normal pancreas development yet critical in progression and tumor biology of pancreas cancer.. Genes Dev.

[15] Ijichi H, Chytil A, Gorska AE, Aakre ME, Fujitani Y, Fujitani S, Wright CV, Moses HL (2006). Aggressive pancreatic ductal adenocarcinoma in mice caused by pancreas-specific blockade of transforming growth factor-beta signaling in cooperation with active Kras expression.. Genes Dev.

[16] Padua D, Massagué J (2009). Roles of TGFbeta in metastasis.. Cell Res.

[17] Langenskiöld M, Holmdahl L, Falk P, Angenete E, Ivarsson ML (2008). Increased TGF-beta 1 protein expression in patients with advanced colorectal cancer.. J Surg Oncol.

[18] Zhao Z, Ma W, Zeng G, Qi D, Ou L, Liang Y (2012). Preoperative serum levels of early prostate cancer antigen (EPCA) predict prostate cancer progression in patients undergoing radical prostatectomy.. Prostate.

[19] Shariat SF, Shalev M, Menesses-Diaz A, Kim IY, Kattan MW, Wheeler TM, Slawin KM (2001). Preoperative plasma levels of transforming growth factor beta(1) (TGF-beta(1)) strongly predict progression in patients undergoing radical prostatectomy.. J Clin Oncol.

[20] Chen J, Yao ZX, Chen JS, Gi YJ, Muñoz NM, Kundra S, Herlong HF, Jeong YS, Goltsov A, Ohshiro K, Mistry NA, Zhang J, Su X (2016). TGF-β/β2-spectrin/CTCF-regulated tumor suppression in human stem cell disorder Beckwith-Wiedemann syndrome.. J Clin Invest.

[21] Chen J, Zaidi S, Rao S, Chen JS, Phan L, Farci P, Su X, Shetty K, White J, Zamboni F, Wu X, Rashid A, Pattabiraman N (2018). Analysis of Genomes and Transcriptomes of Hepatocellular Carcinomas Identifies Mutations and Gene Expression Changes in the Transforming Growth Factor-β Pathway.. Gastroenterology.

[22] Chen J, Mitra A, Li S, Song S, Nguyen BN, Chen JS, Shin JH, Gough NR, Lin P, Obias V, He AR, Yao Z, Malta TM (2020). Targeting the E3 Ubiquitin Ligase PJA1 Enhances Tumor-Suppressing TGFβ Signaling.. Cancer Res.

[23] Malta TM, Sokolov A, Gentles AJ, Burzykowski T, Poisson L, Weinstein JN, Kamińska B, Huelsken J, Omberg L, Gevaert O, Colaprico A, Czerwińska P, Mazurek S, Cancer Genome Atlas Research Network (2018). Machine Learning Identifies Stemness Features Associated with Oncogenic Dedifferentiation.. Cell.

[24] Giannelli G, Villa E, Lahn M (2014). Transforming growth factor-β as a therapeutic target in hepatocellular carcinoma.. Cancer Res.

[25] Rhodes DR, Yu J, Shanker K, Deshpande N, Varambally R, Ghosh D, Barrette T, Pandey A, Chinnaiyan AM (2004). ONCOMINE: a cancer microarray database and integrated data-mining platform.. Neoplasia.

[26] Gao S, Alarcón C, Sapkota G, Rahman S, Chen PY, Goerner N, Macias MJ, Erdjument-Bromage H, Tempst P, Massagué J (2009). Ubiquitin ligase Nedd4L targets activated Smad2/3 to limit TGF-beta signaling.. Mol Cell.

[27] Briones-Orta MA, Levy L, Madsen CD, Das D, Erker Y, Sahai E, Hill CS (2013). Arkadia regulates tumor metastasis by modulation of the TGF-β pathway.. Cancer Res.

[28] Kavsak P, Rasmussen RK, Causing CG, Bonni S, Zhu H, Thomsen GH, Wrana JL (2000). Smad7 binds to Smurf2 to form an E3 ubiquitin ligase that targets the TGF beta receptor for degradation.. Mol Cell.

[29] Komuro A, Imamura T, Saitoh M, Yoshida Y, Yamori T, Miyazono K, Miyazawa K (2004). Negative regulation of transforming growth factor-beta (TGF-beta) signaling by WW domain-containing protein 1 (WWP1).. Oncogene.

[30] Zhu H, Kavsak P, Abdollah S, Wrana JL, Thomsen GH (1999). A SMAD ubiquitin ligase targets the BMP pathway and affects embryonic pattern formation.. Nature.

[31] Guo X, Ramirez A, Waddell DS, Li Z, Liu X, Wang XF (2008). Axin and GSK3- control Smad3 protein stability and modulate TGF- signaling.. Genes Dev.

[32] Wan M, Cao X, Wu Y, Bai S, Wu L, Shi X, Wang N, Cao X (2002). Jab1 antagonizes TGF-beta signaling by inducing Smad4 degradation.. EMBO Rep.

[33] Wan M, Tang Y, Tytler EM, Lu C, Jin B, Vickers SM, Yang L, Shi X, Cao X (2004). Smad4 protein stability is regulated by ubiquitin ligase SCF beta-TrCP1.. J Biol Chem.

[34] Sharma V, Antonacopoulou AG, Tanaka S, Panoutsopoulos AA, Bravou V, Kalofonos HP, Episkopou V (2011). Enhancement of TGF-β signaling responses by the E3 ubiquitin ligase Arkadia provides tumor suppression in colorectal cancer.. Cancer Res.

[35] Massagué J, Blain SW, Lo RS (2000). TGFbeta signaling in growth control, cancer, and heritable disorders.. Cell.

[36] Mullen AC, Orlando DA, Newman JJ, Lovén J, Kumar RM, Bilodeau S, Reddy J, Guenther MG, DeKoter RP, Young RA (2011). Master transcription factors determine cell-type-specific responses to TGF-β signaling.. Cell.

[37] Consalvi S, Brancaccio A, Dall’Agnese A, Puri PL, Palacios D (2017). Praja1 E3 ubiquitin ligase promotes skeletal myogenesis through degradation of EZH2 upon p38α activation.. Nat Commun.

[38] Zoabi M, Sadeh R, de Bie P, Marquez VE, Ciechanover A (2011). PRAJA1 is a ubiquitin ligase for the polycomb repressive complex 2 proteins.. Biochem Biophys Res Commun.

[39] Sasaki A, Masuda Y, Iwai K, Ikeda K, Watanabe K (2002). A RING finger protein Praja1 regulates Dlx5-dependent transcription through its ubiquitin ligase activity for the Dlx/Msx-interacting MAGE/Necdin family protein, Dlxin-1.. J Biol Chem.

[40] Saha T, Vardhini D, Tang Y, Katuri V, Jogunoori W, Volpe EA, Haines D, Sidawy A, Zhou X, Gallicano I, Schlegel R, Mishra B, Mishra L (2006). RING finger-dependent ubiquitination by PRAJA is dependent on TGF-beta and potentially defines the functional status of the tumor suppressor ELF.. Oncogene.

[41] Shin J, Mishra V, Glasgow E, Zaidi S, Chen J, Ohshiro K, Chitti B, Kapadia AA, Rana N, Mishra L, Deng CX, Rao S, Mishra B (2017). PRAJA is overexpressed in glioblastoma and contributes to neural precursor development.. Genes Cancer.

[42] Mitra A, Yan J, Xia X, Zhou S, Chen J, Mishra L, Li S (2017). IL6-mediated inflammatory loop reprograms normal to epithelial-mesenchymal transition
^+^
metastatic cancer stem cells in preneoplastic liver of transforming growth factor beta-deficient β2-spectrin
^+/-^
mice.. Hepatology.

[43] Yao ZX, Jogunoori W, Choufani S, Rashid A, Blake T, Yao W, Kreishman P, Amin R, Sidawy AA, Evans SR, Finegold M, Reddy EP, Mishra B (2010). Epigenetic silencing of beta-spectrin, a TGF-beta signaling/scaffolding protein in a human cancer stem cell disorder: Beckwith-Wiedemann syndrome.. J Biol Chem.

[44] Gingold JA, Zhu D, Lee DF, Kaseb A, Chen J (2018). Genomic Profiling and Metabolic Homeostasis in Primary Liver Cancers.. Trends Mol Med.

[45] Zucman-Rossi J, Villanueva A, Nault JC, Llovet JM (2015). Genetic Landscape and Biomarkers of Hepatocellular Carcinoma.. Gastroenterology.

[46] Schulze K, Imbeaud S, Letouzé E, Alexandrov LB, Calderaro J, Rebouissou S, Couchy G, Meiller C, Shinde J, Soysouvanh F, Calatayud AL, Pinyol R, Pelletier L (2015). Exome sequencing of hepatocellular carcinomas identifies new mutational signatures and potential therapeutic targets.. Nat Genet.

[47] Nault JC, Mallet M, Pilati C, Calderaro J, Bioulac-Sage P, Laurent C, Laurent A, Cherqui D, Balabaud C, Zucman-Rossi J (2013). High frequency of telomerase reverse-transcriptase promoter somatic mutations in hepatocellular carcinoma and preneoplastic lesions.. Nat Commun.

[48] Nault JC, Calderaro J, Di Tommaso L, Balabaud C, Zafrani ES, Bioulac-Sage P, Roncalli M, Zucman-Rossi J (2014). Telomerase reverse transcriptase promoter mutation is an early somatic genetic alteration in the transformation of premalignant nodules in hepatocellular carcinoma on cirrhosis.. Hepatology.

[49] Totoki Y, Tatsuno K, Covington KR, Ueda H, Creighton CJ, Kato M, Tsuji S, Donehower LA, Slagle BL, Nakamura H, Yamamoto S, Shinbrot E, Hama N (2014). Trans-ancestry mutational landscape of hepatocellular carcinoma genomes.. Nat Genet.

[50] Korkut A, Zaidi S, Kanchi RS, Rao S, Gough NR, Schultz A, Li X, Lorenzi PL, Berger AC, Robertson G, Kwong LN, Datto M, Roszik J, Cancer Genome Atlas Research Network (2018). A Pan-Cancer Analysis Reveals High-Frequency Genetic Alterations in Mediators of Signaling by the TGF-β Superfamily.. Cell Syst.

[51] Teixeira AF, Ten Dijke P, Zhu HJ (2020). On-Target Anti-TGF-β Therapies Are Not Succeeding in Clinical Cancer Treatments: What Are Remaining Challenges?. Front Cell Dev Biol.

